# Involvement of Mfn2, Bcl2/Bax signaling and mitochondrial viability in the potential protective effect of Royal jelly against mitochondria-mediated ovarian apoptosis by cisplatin in rats

**DOI:** 10.22038/ijbms.2020.40401.9563

**Published:** 2020-04

**Authors:** Khalid S. Hashem, Asmaa Mohammed M. Hussein Elkelawy, Saber Abd-Allah, Nermeen A. Helmy

**Affiliations:** 1 Department of Biochemistry, Faculty of Veterinary Medicine, Beni-Suef University, Beni-Suef, Egypt; 2 Department of Pharmacology, Faculty of Medicine, Beni-Suef University, Beni-Suef, Egypt; 3 Department of Theriogenology, Faculty of Veterinary Medicine, Beni-Suef University, Beni-Suef, Egypt; 4 Department of Physiology, Faculty of Veterinary Medicine, Beni-Suef University, Beni-Suef, Egypt

**Keywords:** Bax, Bcl2, Mfn2, Mitochondrial viability, Ovaries

## Abstract

**Objective(s):**

The current study aimed to assess cisplatin-mediated ovarian apoptosis in a rat model by Royal jelly (RJ).

**Materials and Methods:**

Thirty female adult albino rats (180-200 g) were divided into three groups (n=10): saline (0.9% NaCl, IP) was given to the control group, the cisplatin group: received (5 mg/kg/once a week IP) for 5 successive weeks, the RJ+Cis. group: received RJ (100 mg/kg/ day PO daily), and Cisplatin (5 mg/kg/once per week IP) for 5 successive weeks. At the end of the experiment, rats were sacrificed and their ovaries were isolated and used for biochemical analysis, molecular investigations and morphometric assessment as well as histological study. Moreover, blood samples were collected for determination of follicle-stimulating hormone (FSH), luteinizing hormone (LH), Estradiol, progesterone and anti-mullerian hormone (AMH).

**Results:**

The current study clarified that RJ given to rats prior to cisplatin significantly increased the ovarian and uterine weights, in addition to follicular count at *P˂*0.05 compared to rats injected only with cisplatin. Moreover, it restored normal ovarian histological structure with a concurrent reduction in FSH, and LH levels, and increased AMH and ovarian hormone concentrations at *P˂*0.05 compared to cisplatin group. Also, RJ decreased the ovarian antioxidant/oxidative imbalance harmonized with significant suppression of inducible nitric oxide synthase and increase of quinone oxidoreductase 1 mRNA expression at *P*˂0.05 compared to cisplatin group.

**Conclusion:**

We concluded that RJ could alleviate mitochondrial-induced ovarian apoptosis caused by cisplatin via increasing anti-apoptotic Bcl2, and diminishing pro-apoptotic Bax with a concomitant increase of Mfn2 mRNA and protein expressions.

## Introduction

The reduced oocyte reserve, ovarian complications and infertility are regular adverse events of several cancer therapeutic approaches such as irradiation and chemotherapy drugs (1, 2). Among these agents, cisplatin [CDDP, cis-diamminedichloroplatinum (II)] is increasingly used in the management of various types of cancer especially bladder, head, neck, and lung cancers (3).

Cisplatin has various adverse effects such as nephrotoxicity, ototoxicity, cardiotoxicity, and hepatotoxicity, and causes gastrointestinal and reproductive dysfunction (4, 5). Many theories explained the mechanism of cisplatin-induced tissue damage including, chemical bonding and crosslinking of DNA, which induces cell apoptosis (6, 7), induction of the cell membrane lipid peroxidation (8), disturbance of mitochondrial function (9), and protein synthesis inhibition (10). Moreover, increasing production of reactive oxygen species (ROS) and suppression of antioxidant capacity are the most detectable adverse impacts of cisplatin (11-13). These toxic effects extend also to involve the ovarian tissue, and the follicular granulosa cells, causing infertility due to acute and chronic ovarian failure (14). Exhaustion of the ovarian reserve and the resultant premature ovarian failure attributed to cisplatin-induced ovarian dysfunction may lead to disturbance in the menstrual cycle and damage of primordial follicles due to apoptosis in human females (15, 16). Biochemical variations of follicle-stimulating hormone (FSH), luteinizing hormone (LH), Estradiol 2 (E2) and anti-mullerian hormone (AMH) levels are considered a direct indicator for ovarian damage and reduced follicular reserve following chemotherapy (17-19).

Mitochondria, the main drive-provider to the cell, play an essential role in the process of cellular apoptosis. It controls cellular metabolism, cell cycle, and signal transition (20). Many studies proved that decreased mitochondrial viability is involved in the pathogenesis of ovarian damage and apoptosis (21). Mitochondrial-induced granular cell disturbance and disruption of oocyte development could be confined to the ability of mitochondria to alter ATP production, increase the production of reactive oxygen species (ROS), and then modify granular cell function as well as the development of oocytes. This extends even to the point of inducing cell death (22). 

Previous studies have clarified that Bcl-2 acts on the mitochondria. It has obvious anti-apoptotic effect attributed to its ability to modify the intracellular Ca^2+ ^balance, which is a second messenger involved in regulating cell survival and apoptosis. The release of Ca^2+^ to the cytoplasm and mitochondria from endoplasmic reticulum (ER) is facilitated by inositol 1,4,5-trisphosphate receptor (IP3R) that resulted in mitochondrial Ca^2+^overload (23). Stary *et al.*, reported that voltage-dependent anion channel 1 (VDAC1), an outer mitochondrial membrane protein, controls Ca^2+^ entry into the mitochondria (24). This Ca^2+ ^overload opens the mitochondrial permeability transition pore (mPTP) and causes the loss of mitochondrial membrane potential (Δψm) that facilitates the mitochondrial-mediated programmed cell death (25). The anti-apoptotic mechanism of Bcl2 could be described by its ability to interact with IP3R preventing Ca^2+ ^transfer to the cytoplasm and mitochondria (26) and also binding Bcl2 with VDAC1 to prevent the Ca^2+ ^entry to mitochondria and thus preventing mitochondrial-induced cellular apoptosis (27). Moreover, Yuan *et al.* reported that Bcl2 has the ability to increase the expression of fusion mitochondrial proteins Mitofusin 2 (Mfn2) that could be another mechanism explaining the anti-apoptotic effect of Bcl2 (28). Mfn2 is a trans-membrane GTPase protein, which presents not only in the outer mitochondrial membrane but also in mitochondrial-associated membranes. This protein, beside its ability to control ER morphology, is also incorporated in mitochondrial fusion and controls the transfer of calcium from the ER to mitochondria (29-31). Furthermore, Mfn2 exerts a vital role in metabolic homeostasis, energy metabolism, mitochondrial morphology, ER stress, signal transduction (32) and mitochondrial integrity thus favors the survival of the cell (28). Nearby, it has an indispensable role in the synthesis of the blastocyst and early embryonic development (33, 34).

Royal jelly (RJ) is a white and thick jelly-like substance, which is a type of the worker bees’ hypopharyngeal and mandibular gland secretion components (35). It possesses many beneficial effects including antitumor, and antioxidant activities in addition to improvement of menopausal symptoms in different animal models (36, 37). The most elevated constituents of RJ are water (50% to 60%), proteins (18%), carbohydrates (15%), lipids (3%–6%), mineral salts (1.5%), and vitamins (38). Also, RJ contains numerous bioactive mixes including fatty acid, proteins, adenosine monophosphate (AMP), polyphenols, and hormones such as testosterone, progesterone, prolactin, and also estradiol (39). Furthermore, the antioxidant role and free radicals scavenging effect of RJ (40) could be attributed to the flavonoids, phenolic compounds (41), and free amino acids such as aspartic acid, cysteine, cystine, tyrosine, glycine, lysine, leucine, valine, and isoleucine (42). In addition, RJ possesses an immune-stimulatory effect due to the presence of 10-hydroxy-2-decenoic acid (HAD) (43). 

Ibrahim *et al.* described that RJ exerts a protective role against cisplatin-induced kidney injuries via suppression of fibrogenic factors, α smooth muscle actin (α -SMA) and transforming growth factor β1(TGF- β1) (44). Recently, mitochondria is considered as a target of RJ components (45), particularly leucine (46), and 10-hydroxy-2-decenoic acid (47), which is the exclusive fatty acid that induces the activation of AMP-activated protein kinase (AMPK) that is considered as the most essential mediator of the mitochondrial biogenesis in many tissues like skeletal muscles (48).

For these reasons, we suggest that RJ might apply a protective role on mitochondrial-mediated ovarian apoptosis caused by cisplatin. The protective effects of RJ have been previously studied, but the suggested protective mechanism of RJ on mitochondria-induced ovarian toxicity by cisplatin has not been developed yet. Therefore, this study aimed to design a rat model for evaluation of the potential protective effects of RJ on mitochondrial-mediated ovarian apoptosis by cisplatin. 

## Materials and Methods

***Chemicals***

Cisplatin was obtained from Sigma-Aldrich Corporation (St. Louis, MO), CASE number: 15663-27-1. RJ soft capsules were purchased from Pharco Pharmaceuticals Co. (Alexandria, Egypt). All chemicals used in this experiment were of analytical grade. 

***Animals***

In this study, 30 female adult albino rats with average weight of 180- 200 g were used. The animals were accommodated at the animal house, Faculty of Medicine and were provided with a standard pallet diet and water *ad libitum* and kept under conditions of adequate ventilation and temperature. Animals were divided into four groups (n=10) including:

- Control: Rats given normal saline (0.9% NaCl 1 ml IP)

- Cisplatin: Rats received cisplatin (5 mg/kg/once a week IP) for 5 successive weeks (49). 

- Cis and RJ: Rats were given RJ (100 mg/kg/ day PO**) **(50) daily for 5 consecutive weeks half an hour prior to cisplatin (5 mg/kg/once a week IP) for 5 consecutive weeks. 

All animal dealings were constructed following the strict guidelines assigned by Institutional Animal Care and Use Committee (IACUC-Beni-Suef University) at Beni-Suef University, faculty of veterinary medicine, Beni-Suef, Egypt.

***Sampling***

*Specimen collection*

At the end of the experiment, all animals were sacrificed under light anesthesia. Ovaries were isolated, weighted by using single-pan electronic balance (Leyte, Guangdong, China (Mainland)) and divided into three parts. The first one was dissected for morphometric analysis and histological study, the second part was prepared for biochemical evaluation and the third part was used for molecular assessment. In addition, the collected blood samples were subjected to centrifugation to obtain serum that was kept frozen and used for hormonal analysis (determination of serum FSH, LH, estradiol, progesterone and AMH)

***Morphometric analysis of ovarian tissue and quantification study of folliculogenesis***

For fixation of the ovary and uterine horn, Bouin’s fluid was used. Graded dehydration of the tissue was performed by 70 to 100% alcohol in successive steps. Xylene was used as the clearing agent. The tissues were embedded in paraffin (58.6°C). Sections of paraffin blocks were cut by a rotatory microtome (CRAFTEK, China) into 5 µm-thick paraffin sections and were processed to prepare for hematoxylin and eosin (H&E) staining (51) and were then inspected under a microscope (Sanli,  China Mainland). The quantification study of folliculogenesis was assessed consistent with Patil *et al.* (52) with minor modifications. According to follicles diameters and morphologies, they were classified as follow:

Class I: Small pre-antral follicle (SPAF) (<94 µm);

Class II: Large pre-antral follicle (LPAF) (94–260 µm);

Class III: Small antral follicle (SAF) (261–350 µm);

Class IV: Medium antral follicle (MAF) (351–430 µm);

Class V: Large antral follicle (LAF) (431–490 µm);

Class VI: Graafian follicles (GF) (<491 µm).

For histological evaluation, method of Li *et al.* was used (53) with some modifications. The histological sections, stained with H&E, and were inspected for the existence of vascular congestion, hemorrhage, follicular degeneration, hyalinosis and interstitial edema. According to the obtained histological findings, changes were scored from 0 to 3, where 0 indicates no pathological changes of the ovary, while 1, 2 and 3 represent pathological changes of <33%, 33–66% and >66%, respectively. The scores for each parameter were calculated and the total scores were obtained and presented as means±SEM.

***Measurement of oxidative/antioxidant parameters***

For tissue homogenate preparation, 0.5 gram of ovarian tissue was homogenized in 5 ml saline (NaCl 0.9%) by using homogenizer (Ortoalresa, Spain). The homogenates were centrifuged at 1000 X g for 15 min. The supernatant was collected in Eppendorf tubes that were kept in the deep freezer (at -80 ^°^C) for further biochemical evaluation according to the directions of the biochemical assay kits. 

***Biochemical assays (Colorimetric method)***

The supernatant of ovarian tissue homogenates were used for measurement of reduced glutathione (GSH), catalase (CAT), superoxide dismutase (SOD), total antioxidant capacity (TAC) and total oxidative stress (TOS) by spectrophotometry (UV-1700; Shimadzu Corporation, Kyoto, Japan). All colorimetric kits were obtained from Biodiagnostic Company for chemicals, Egypt.

GSH concentration was measured according to the method of Beutler *et al.* ( 54), CAT was determined according to the method of Aebi (55), SOD was measured according to the method of Nishikimi *et al.* (56)**, **and TAC was determined by the reaction of antioxidants in the sample with a distinct amount of exogenously provide hydrogen peroxide (H_2_O_2_). The antioxidants in the sample abolish a definite amount of the provided hydrogen peroxide. The residual H_2_O_2_ is determined colorimetrically (57).

Determination of TOS level was performed using a colorimetric measurement method, which was described by Erel (58), and the oxidative stress index (OSI) was calculated as the percentage ratio of TOS to TAC levels according to the following formula: 

OSI (arbitrary unit)=TOS (micromolar H2O2 equivalent/liter)/TAC (micromolar equivalent/liter) (59).

***Ovarian mitochondria enriched fraction***

0.5 gram of ovarian homogenate was kept on ice and added to isolation medium (10 mM HEPES buffer pH 7.0 containing 220 mM mannitol, 68 mM sucrose, 10 mM KCl and 0.1% serum albumin) in a ratio 1:10. Centrifugation was performed for 10 min at 1000 X g, and then the supernatant was re-centrifuged at 11,500 X g for 10 min. The supernatant was discarded, and the pellet was re-suspended in the isolation medium but without albumin (60).

***Assessment of ovarian mitochondrial function***

Mitochondrial function was evaluated using MTT reduction assay. This assay is linked to the ability of the mitochondrial dehydrogenases to metabolize 3-(4,5-dimethylthiazol-2-yl)-2,5-diphenyltetrazolium bromide (MTT) to formazan, a reaction that occurs if the mitochondrial preparation is functionally intact. 

***DNA fragmentation %***

The ovarian tissues were added to 0.5 ml lysis buffer (10 mM Tris-HCl (PH 8), 1 mM EDTA, 0.2% triton X 100), then centrifuged at 10000 rpm for 20 min at 4 ^°^C. Supernatant and sediment were collected in separate Eppendorf tubes. 0.5 ml of 25 % Trichloroacetic acid was added to the sediment and supernatant and incubated at 4 ^°^C for 24 hr. The samples were centrifuged for 20 minutes at 10000 rpm at 4 ^°^C, and then incubate at 83 ^°^C for 20 min. Subsequently, 160 µl of Diphenylamine (DPA) solution (150 gram DPA in 10 ml glacial acetic acid, 150 µl sulphuric acid and 50 µl acetaldehyde (16 mg/ ml)) was added and incubated at room temperature for 24 hr (61). The portion of fragmented DNA was calculated from the absorbance reading at 600 nm using the following formula:

$\mathrm{Fragmented DNA\%}=\frac{\mathrm{OD of Supernatant}}{\mathrm{OD of supernantant}+\mathrm{OD of standar}}\times 100$

***Detection of iNOS, NQO1 and Mfn2 mRNA expression by RT-PCR***

Based on the instruction of the kit and by using RNeasy Purification Reagent (Qiagen, Valencia, CA), the total RNA was isolated from ovarian homogenates. The concentration of RNA was measured using a UV spectrophotometer. Afterwards, mRNA expressions of inducible nitric oxide synthase (iNOS), quinone oxidoreductase 1(NQO1) and Mfn2 were evaluated by RT-PCR.

***cDNA synthesis***

Five microgram RNA was reverse transcribed and denatured by using oligonucleotide (dT) 18 primer (final concentration, 0.2 mM) and keeping at 70 ^°^C for 2 min, respectively. Denatured RNA was kept on ice and in the reverse transcription mixture containing 50 mM KCl, 50 mM Tris HCl (pH 8.3), 0.5 mM of deoxyribonucleotide triphosphate (dNTP), 3 mM MgCl2, 1 U/ml RNase inhibitor, and 200 units of murine leukemia virus reverse transcriptase. The reaction tube was exposed at 42 ^°^C for 1 hr, followed by heating to 92 ^°^C to stop the reaction.

***Real-time quantitative polymerase chain reaction***

Five microliter of the first-strand cDNA was used in a total volume of 25 μl, containing 12.5 μl 2x SYBR Green PCR Master Mix (Applied Biosystems, Foster City, CA, USA) and 200 ng of each primer as shown in Table 1. PCR program was 1 cycle at 95 ^°^C/ 10 min, 94 ^°^C/ 15 sec and 40 cycles at 60 ^°^C/ 1 min, by using step one plus Real Time PCR system (Applied Biosystems). Data analysis was performed by the ABI Prism 7500 sequence detection system software and quantified using the v 1.7 Sequence Detection Software from PE Biosystems (Foster City, CA). Relative expression of studied genes was calculated using the comparative threshold cycle method. All values were normalized to the beta actin genes, and all these steps were described by Livak and Schmittgen (62).

***Western blotting analysis***

Thirty microgram proteins were separated by SDS-PAGE, and moved to PVDF membranes (Invitrogen, USA). 5% non-fat milk was used for 1 hr at 37 ^°^C to block the non-specific binding. Overnight hybridization of nitrocellulose membranes was performed with the rabbit polyclonal anti-Mfn2 antibody (Abcam, USA), the rabbit polyclonal anti-Bcl-2 antibody (Cell Signaling Technology, USA), the rabbit polyclonal anti-Bax antibody (Cell Signaling Technology, USA), and the rabbit polyclonal anti-β-actin antibody (Santa cruz Biotechnology Inc, USA) in the Primary Antibody Dilution Buffer at 4 ^°^C. After four times washing of the bands with TBS-T, each time for 10 min at 37 ^°^C, the membranes were incubated with goat anti-rabbit horseradish peroxidase (HRP)-conjugated secondary antibody for 1 hr at 37 ^°^C (AntGene, USA). Finally, the immune reactive bands were detected by the enhanced chemiluminescence system (Beyotime Institute of Biotechnology, China). The intensity of the band was quantified by densitometry using the Quantity One 4.62 analysis software, and all results were normalized to β-actin signal intensity.

***Statistical analysis***

All results were analyzed using SPSS 18.0 (SPSS, Inc., Chicago, IL, USA). The obtained results were expressed as means±SD. Significant differences between means were verified by one-way ANOVA. The calculated data were determined to be significant if the *P*<0.05.

## Results

***Ovarian and uterine weight***

Results in Table 2 showed that administration of cisplatin significantly (*P*<0.001) reduced ovarian (50.1%) and uterine weight (43.7%) in comparison with the control group. On the other side, RJ significantly (*P*<0.001) increased ovarian (89.5%) and uterine weight (75.4%) compared to the animal group injected with cisplatin (Table 2). 

***Ovarian morphometric analysis and quantitation of folliculogenesis***

Ovaries subjected to cisplatin showed a significant (*P*<0.001) decrease of small preantral follicle (SPAF), large preantral follicle (LPAF), small antral follicle (SAF), medium antral follicle (MAF), large antral follicle (LAF) and Graafian follicles (GF) and a significant (*P*<0.01) decrease of corpus luteum number when compared to control group. Moreover, injection of cisplatin caused a significant (*P*<0.001) increase in the number of atretic follicles compared to control group. On the contrary, ovaries exposed to RJ in RJ+Cis. group showed a significant (*P*<0.001) increase in the number of SPAF, LPAF, SAF, MAF, AF (*P*<0.01) and GF(*P*<0.01) as well as corpusluteum when compared to cisplatin group (Table 3).

***Histological changes of ovaries***

**Figure 1:**
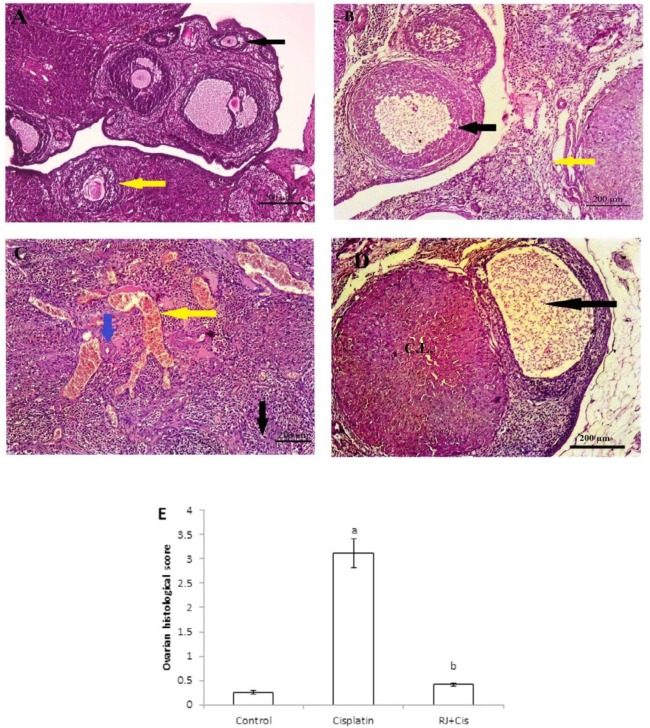
Representative photomicrographs of H&E-stained sections of rat ovaries in each experimental group

The alterations of ovarian histology in different groups are presented in Figure 1. The histological sections stained with H&E revealed that those of control had normal ovarian architecture with no considerable pathologic alteration. Normal ovarian follicles in various stages of development were observed in the ovarian cortex (Figure 1A). However, ovaries of cisplatin group showed follicular degeneration (black arrow), interstitial edema (yellow arrow) (Figure 1B), marked vascular congestion (yellow arrow), hyalinosis (blue arrow) as well as stromal edema (black arrow) (Figure 1C). Administration of RJ with cisplatin in RJ+Cis. group restored the normal structure of the ovarian tissue with no significant difference when compared to the control group (Figure 1D). 

***Serum gonadotropins, female sex hormones and AMH***

Cisplatin administration caused a significant increase of FSH (*P*<0.001) and LH (*P*<0.01) concentrations with respect to control group. In addition, it significantly (*P*<0.001) decreased estradiol (E2), progesterone and AMH concentration compared to control group. On the other side, RJ administration prior to cisplatin in RJ+Cis group significantly reduced FSH (*P*<0.001) and LH (*P*<0.01) concentrations with a simultaneous significant (*P*<0.001) increase of E2, progesterone and AMH concentration compared to cisplatin group (Table 4). 

***Ovarian antioxidant/oxidative redox***

**Figure 2:**
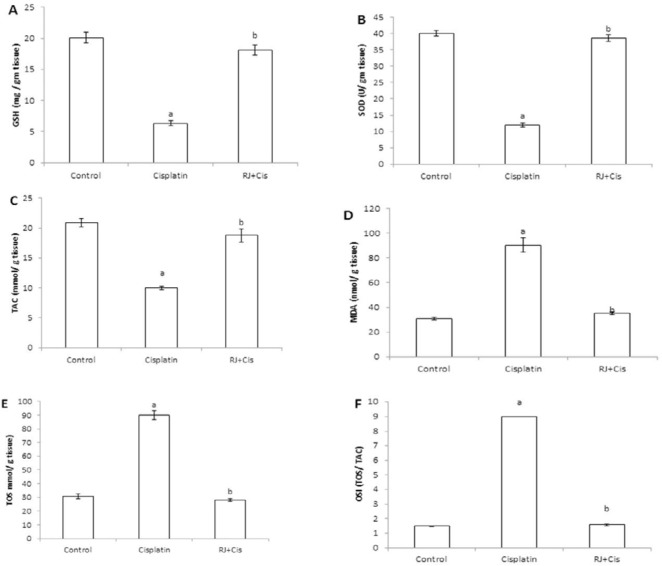
Antioxidant activity assays in ovarian tissues of all experimental groups

Data in Figure 2 showed that cisplatin administration significantly reduced ovarian GSH concentration (*P*<0.001), SOD activity (*P*<0.001) and TAC (*P*<0.001) compared to control rats. Added to that, cisplatin significantly increased MDA (*P*<0.001), TOS (*P*<0.001) and OSI (*P*<0.001) compared to control group. However, RJ significantly restored the ovarian antioxidant activity, which was demonstrated by the significant increase of ovarian GSH concentration (*P*<0.001), SOD activity (*P*<0.001) and TAC (*P*<0.001) compared to cisplatin-treated rats. In addition, RJ significantly reduced the ovarian MDA (*P*<0.001), TOS (*P*<0.001) and OSI (*P*<0.001) compared to cisplatin group. 

***Ovarian iNOS, NQO1, mitochondrial viability and DNA fragmentation %***

**Figure 3:**
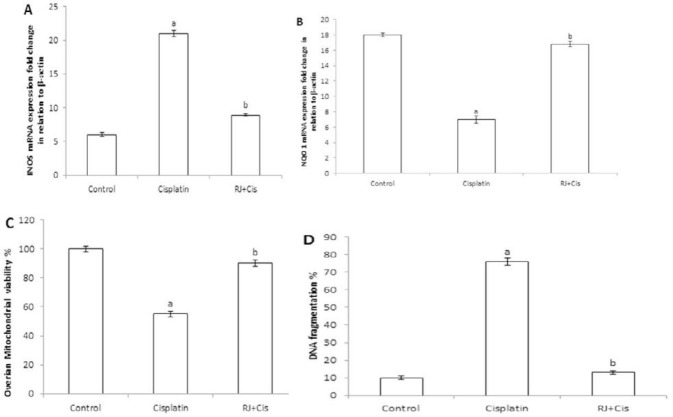
Estimation of ovarian iNOS, NQO1, mitochondrial viability % and DNA fragmentation %

Results represented in Figure 3 showed that cisplatin administration significantly increased ovarian iNOS (*P*<0.001), and reduced NQO1 (*P*<0.001), mitochondrial viability (*P*<0.001) and DNA fragmentation % (*P*<0.001) as compared to control group. RJ significantly decreased iNOS (*P*<0.001), and increased NQO1 (*P*<0.001), mitochondrial viability (*P*<0.001) and decreased DNA fragmentation % (*P*<0.001) compared to cisplatin group. 

***Ovarian Mfn2, Bcl2 and Bax mRNA expressions and protein concentrations***

**Figure 4:**
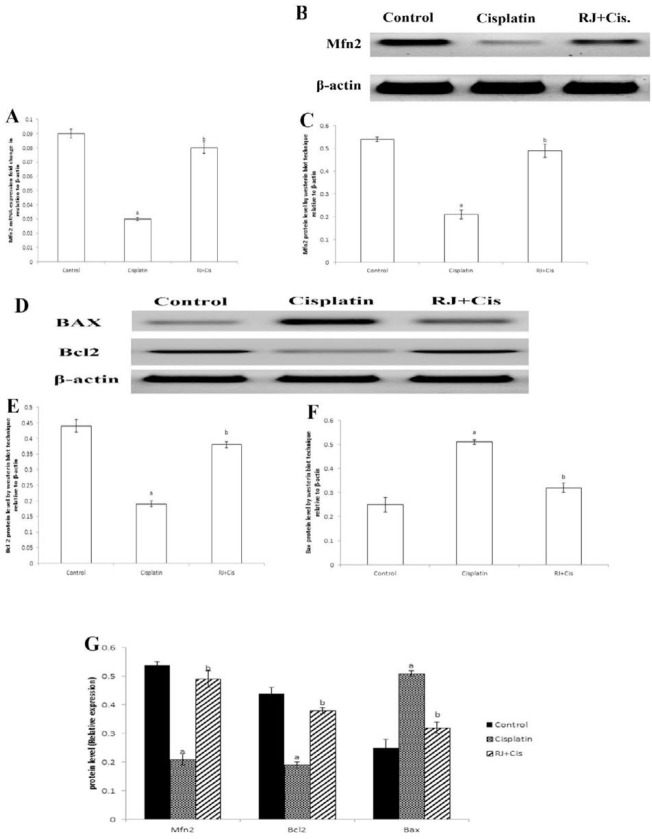
Ovarian Mfn2 mRNA expression, Mfn2, Bcl2 and Bax protein levels

Results in Figure 4 showed that cisplatin significantly decreased mRNA expressions of Mfn2 (*P*<0.001), and Bcl2 and protein concentrations (*P*<0.001) and significantly increased Bax mRNA expression and protein concentration (*P*<0.001) compared to control group. RJ restored Mfn2 mRNA expression and protein and re-established ovarian Bcl2/ Bax mRNA and proteins (*P*<0.001) compared to cisplatin group.

## Discussion

Many previous studies highly suggested the use of protective compounds of plant source with chemotherapeutic agents to enhance their efficacy and reduce their toxic effects (63, 64). The current study intended to postulate that RJ could ameliorate cisplatin-induced ovarian oxidative stress and apoptosis.

Chemotherapeutic medicines impair fertility as they severely disturb the ovarian activities, development and hormonal balance (65). One of the most commonly used chemotherapies is cisplatin. It is used in the management of several types of tumors. However, it was reported that cisplatin caused serious unwanted effects on kidneys (66), neurons (67), stomach (68) and reproductive organs (68). Han *et al. *described that cisplatin increased the prevalence of premature ovarian function disturbance in humans (69).

Our generated data demonstrated that injection of cisplatin caused serious ovarian damage, which was indicated by the reduction of ovarian and uterine weight, decrease in the follicular count and increase in the number of atretic follicles. These results are in harmony with those of Ozdamar *et al. *(70) who reported that chemotherapies result in follicular damage followed by ovarian dysfunction. Regarding cisplatin, it was reported that its administration induced follicular and ovarian damage (70, 71). According to the results of the existing study, histopathological examination of ovaries, in cisplatin-treated animals, revealed stromal edema, severe congestion, hyalinosis and marked degeneration of follicles in cisplatin-treated rats. The findings were similar to those obtained by Altuner *et al. *(72).

Our results indicated that cisplatin decreased E2 and progesterone concentrations due to the extensive follicular damage, which can result in the loss of ovarian steroid hormones (73). A simultaneous increase of serum FSH and LH concentrations was noticed and harmonized with diminished ovarian negative feedback due to exhaustion of ovarian follicles (74). Rats injected by cisplatin showed a significant decrease of AMH, which is considered one of the most delicate biomarkers of ovarian damage and reserve, because serum AMH concentration decreases with low ovarian reserve and follicular destruction (71, 75). This result is in agreement with Yeh *et al.* who clarified that cisplatin decreases serum AMH levels in animals (76).

Many previous studies reported that free radicals overproduction and antioxidants depletion are implicated in cisplatin-induced toxicity and tissue damage (72, 77). This comes in concurrence with the results of the existing study that demonstrated low ovarian antioxidants, which were manifested by the decrease of GSH, SOD activity and TAC level coincide with cisplatin administration. A synchronized increase of lipid peroxidation was indicated by the increase of ovarian MDA concentration and OSI. These results agreed with other reports, which stated that cisplatin toxicity is closely related to increased lipid peroxidation (78, 79). Also, MDA can disturb the permeability and fluidity of the cell membrane by interrupting ionic transport and cellular enzymatic activity (80-82).

iNOS is involved in nitric oxide (NO) production and implicated in the initial step of toxicity under oxidative stress. Too much No react with superoxide anion to generate peroxynitrite radical that causes cellular injury by oxidizing cellular macromolecules as GSH, proteins, lipids and DNA to produce the peroxynitrite radical, which causes cell injury (83). In addition, excess NO depletes intracellular GSH, thereby augmenting the sensitivity to oxidative stress (84). Indeed, ovarian iNOS mRNA expression significantly increased by cisplatin confirming the induction of ovarian oxidative stress as mentioned by Krishna *et al.* (85). Once activated, iNOS mRNA expression increases the production of NO, which implicated in cisplatin-mediated ovarian function impairment (86). Nearby the ability of cisplatin to induce many ROS, it inhibits the ovarian antioxidant defense elements. This effect was obvious in our results and manifested by the significant reduction of ovarian NQO1 mRNA expression in cisplatin-treated rats. 

Besides the function of mitochondria as the energy house of the cell (87), the mitochondrial membranes are essential sites for steroidogenesis in granulosa cells (88). Mitochondria are the target organelle for the disruption of cellular antioxidant/ oxidative dynamic equilibrium state (87). Oxidative stress-induced mitochondrial damage impairs steroidogenesis in granulosa cells and inhibits steroidogenic enzymes and a mitochondrial carrier protein (StAR protein), which possess a pivotal role in the transport of cholesterol into luteal cells mitochondria (88). In our study, cisplatin administration decreased the ovarian mitochondrial viability % in agreement with Chen *et al*. (14) and increased ovarian DNA fragmentation % in line with Park *et al.* (89) who informed that excessive ROS is associated with mitochondrial dysfunction and DNA fragmentation %.

Apoptosis is defined as a programmable cellular death with certain metabolic and morphological alterations, excessive nuclear damage, chromatin condensation and the stimulation of specific markers (90, 91). Consequently, the induction of apoptosis is considered as an important pathway in ovarian toxicity and damage. Acquired data in this study showed a significant alteration of ovarian anti-apoptotic/ apoptotic markers following cisplatin treatment. This could represent the cytotoxic effect of cisplatin that is facilitated via the apoptotic pathway. Upon cisplatin treatment, the level of Bcl2 was diminished and that of Bax was augmented. These variations in protein expression changed the permeability of mitochondrial membrane and viability causing the discharge of cytochrome C into the cytosol, which results in the stimulation of the adaptor molecule apoptotic proteaseactivating factor 1 (Apaf1), and producing the apoptosome complex. Apaf1 then cleaves the preform of caspase9 to initiate the caspase cascade, resulting in apoptosis (92). Thus, mitochondrial facilitated apoptosis comprises induction of Bax, suppression of Bcl2, disturbance of mitochondrial viability, and initiation of the caspase cascade. 

The reduced expression of Mfn2 can cause mitochondrial dysfunction and damage, thus provoking the cellular apoptosis (34), oocyte and follicular developmental disorders (93). In our investigation, we clarified that cisplatin administration caused a prominent suppression in ovarian Mfn2 mRNA and protein. This finding comes in line with Chen *et al. *(14) who revealed that expression of Mfn2 was reduced in the ovarian tissues of premature ovarian failure (POF) induced by cisplatin, and that reality might be a mechanism engaged with both ovarian mitochondrial damage and an increase in ovarian apoptosis. Also, many previous studies showed that the lower expression of Mfn2 could aggravate the stress of the ER that results in the apoptosis of granulosa cells as well as inhibiting steroids production and secretion (94, 95). 

Based on the previously discussed data, oxidative stress and apoptosis are potent mechanisms of cisplatin-induced ovarian damage. So, the use of RJ that has a powerful antioxidant, and anti-apoptotic effect (96, 97) is a logic approach. Our results indicated that RJ protected the ovaries from the damaging outcome of oxidative stress mediated by cisplatin. The protective effect of RJ is manifested by increasing both ovarian and uterine weights, the follicular count, decreasing the atretic follicles and restoring normal histological structure of ovaries in RJ-pretreated rats compared to cisplatin-given rats. In addition, RJ significantly increased estrogen and progesterone level. Previous studies suggested that reduction of FSH and LH concentration in RJ-pretreated rats is a direct sequence of increasing ovarian hormones and the number of ovarian follicles (36),(98). Moreover, Kamakura stated that Royalactin, a 57-kDa protein in RJ, increases the ovarian growth (99), ovulation rate and progesterone levels in luteal phase (100) through epidermal growth factor receptor-facilitated signaling pathway.

The antioxidant effect of RJ is specified by the prominent increase of GSH, SOD, and TAC levels and the reduction of MDA, TOS and OSI that come in harmony with Karadeniz, *et al.* who demonstrated that RJ has a powerful antioxidant effect and reduces cisplatin-induced lipid peroxidation in kidney tissue (96,101). Proportionate to our outcomes,  You *et al.* conveyed that RJ could improve tissue damage caused by excessive NO by reducing iNOS mRNA expression (102). Interestingly, RJ has a direct stimulatory effect on NQO1, which catalyzes two-electron reduction and reclamation of quinones and its derivatives, guarding cells from oxidative stress, and redox cycling (103). NQO1 preserves ubiquinone (co-enzyme Q) and α- tocopherol quinine in their reduced active state, which are two fundamental lipid-soluble antioxidants (104,105). Besides the antioxidant effect of RJ, it possesses a potent protective effect against mitochondria-induced ovarian apoptosis. The anti-apoptotic effect of RJ is detected by increased expression of Bcl2 and suppression of Bax in ovarian tissue. In the current study, we hypothesized that RJ targets Mfn2 to alleviate cisplatin-induced ovarian apoptosis, and this hypothesis agreed with Luo *et al. *(32) who concluded that Mfn2 can be used as a novel target in the treatment of ovarian stress and damage. RJ increased both Mfn2 mRNA expression and protein in ovarian tissue compared to cisplatin-treated rats. The activation of Mfn2 re-established the mitochondrial membrane permeability and integrity (106,107).

Mfn2 is widely expressed in the ovarian granular cells, follicular fluid, inner theca cells, corpus luteum and ovarian stroma, but rarely expressed in the outer theca cells. Mfn2 has the ability to inhibit the development of programmed cell death by suppressing the release of cytochrome C mediated by Bax protein, and relieving the radicals-induced cellular damage (34, 108). The re-establishment of ovarian Bcl2/ Bax levels and keeping cytochrome C inside the mitochondria prevent the activation of Apaf1, then no activation of caspase cascade and finally, no apoptotic reaction can take place. 

**Table 1:** Primer sequences used for RT-PCR

**Gene name**	**Primer sequence**
**iNOS**	**Forward(5′-3′)** **:** **GACCAGAAACTGTCTCACCTG** **Reverse(5′-3′)** **:** **CGAACATCGAACGTCTCACA**
**NQO1**	**Forward (5′-3′): -AGGCTGGTTTGAGCGAGT** **Reverse (5′-3′): ATTGAATTCGGGCGTCTGCTG**
**Mfn2**	**Forward primer (5′-3′): CTTGAAGACACCCACAG-GAACA** ** Reverse primer (5′-3′): GGCCAGCACTTCGCTGATAC**
**-actin**	**Forward primer (5′-3′): GGGAAATCGTGCGTGACATT** **Reverse primer (5′-3′): GCGGCAGTGGCCATCTC**

(iNOS): Inducible nitric oxide synthase, (NQO1): Quinone oxidoreductase 1, (Mfn2): Mitofusin 2

**Table 2:** Effect of Royal jelly on ovarian and uterine weight in cisplatin-treated female rats

	**Ovarian ** **weight in pair** ** (mg)**	**Uterine weight (mg) **
**Control**	40.3 ± 0.22	142.28 ±. 4.46
**Cisplatin**	20.12± 0.5^a^	80.12± 1.28^a^
**RJ + Cis. **	38.12 ± 0.92^b^	140.6 ± 2.42^b^

Values were expressed as mean±SD. RJ mean Royal jelly and Cis. Means cisplatin.

a: express a significant difference when compared to the control group. b: indicates a significant difference when compared to cisplatin group

**Table 3:** Effect of Royal jelly on ovarian morphometric analysis and quantitation of folliculogenesis in cisplatin-treated female rats

	**Follicular Counts**							
	**SPAF**	**LPAF**	**SAF**	**MAF**	**LAF**	**GF**	**Corpus luteum**	**Atretic follicles**
**Control**	26.40±1.4	20.50± 2.4	16.50± 1.2	12.8± 1.6	9.62± 2.2	7.2±1.4	3.8 ±1.2	2.8± 0.8
**Cisplatin**	3.12± 0.6^a^	1.34±0.4^a^	2±0.7^ a^	2± 0.5^a^	1± 0.3^a^	1.1±0.2^ a^	1±0.2^ a^	10.17±0.9^ a^
**RJ+Cis.**	25.1± 2^b^	18.2±0.6^ b^	14.1±0.6^b^	11.2± 0.5^ b^	8.1± 0.2^ b^	5.1±0.3^ b^	2±0.4^b^	3.17± 0.6b

Values were expressed as mean±SD. Cis. refers to cisplatin. (RJ): Royal jelly, (SPAF): Small preantral follicle, (LPAF): Large preantral follicle, (SAF): Small antral follicle, (MAF): Medium antral follicle, (LAF): Large antral follicle (LAF) and (GF) Graafian follicles

a: indicates a significant difference (*P<*0.05) when compared to the control group. b: indicates a significant difference (*P<*0.05) when compared to the cisplatin group

A) Light microscopy of the ovarian tissue with developing follicles in many different stages in the control group. B) Cisplatin group showed a severe histopathologic injury than in other groups, follicular degeneration (black arrow), interstitial edema (yellow arrow). C) A significant marked vascular congestion (yellow arrow), hyalinosis (blue arrow) as well as stromal edema (black arrow) in cisplatin group when compared to the control group. D) Administration of Royal jelly (RJ) with cisplatin in RJ+Cis. group restored the normal structure of the ovarian tissue with no significant difference from control group. E) Quantification of histopathological scores in the ovarian sections from the experimental groups where data were expressed as means±SD, a indicates a significant difference when compared to control group at *P˂*0.05 and b: indicates a significant difference when compared to cisplatin group (*P˂*0.05)

**Table 4:** Effect of Royal jelly on serum gonadotropins, female sex hormones and anti-mullerian hormone in cisplatin treated female rats

**Group**	**FSH (ng/ml)**	**LH (ng/ml)**	** (E2) (ng/ml)**	**Progesterone (ng/ml)**	**AMH (ng/ml)**
**Control**	8.22 ±1.23	4.11 ± 0.65	70.98 ± 1.4	40.98 ± 0.9	9.44 ± 0.66
**Cisplatin**	19.98 ± 0.98^a^	9.2 ± 1.09^ a^	20.65 ± 0.9^ a^	12.09 ± 0.32^ a^	4.76 ± 0.31^ a^
**RJ+Cis**	9.65 ± 0.45^ b^	5.25 ± 0.45^b^	75.12 ± 1.4^b^	35.12 ± 1.07^ b^	8.21 ± 0.28^b^

Values were expressed as mean±SD. (RJ): Royal jelly, FSH: Follicular stimulating hormone, (LH): Luteinizing hormone, (E2): Estradiol 2, (AMH): Anti-mullerian hormone

a: indicates a significant difference (*P<*0.05) when compared to the control group. b: indicates a significant difference (*P<*0.05) when compared to the cisplatin group

A) glutathione (GSH) levels, B) superoxidedismutase (SOD) levels, C) total antioxidant capacity (TAC), D) malondialdehyde (MDA) levels, E) total oxidative stress (TOS) and F) oxidative stress index (OSI). The data represent means±SD (n=10/group). Cisplatin administration significantly reduced ovarian GSH concentration (*P<*0.001), SOD activity (*P<*0.001) and TAC (*P<*0.001) compared to control rats. Added to that, cisplatin significantly increased MDA (*P<*0.001), TOS (*P<*0.001) and OSI (*P<*0.001) compared to control group. Royal jelly (RJ) significantly restored the ovarian antioxidant activity, which was indicated by the significant increase of ovarian GSH concentration (*P<*0.001), SOD activity (*P<*0.001) and TAC (*P<*0.001) compared to cisplatin-treated rats. Also, RJ significantly reduced the ovarian MDA (*P<*0.001), TOS (*P<*0.001) and OSI (*P<*0.001) compared to cisplatin group. a: indicates a significant difference when compared to the control group at *P<*0.05, b: indicates a significant difference when compared to cisplatin group at *P<*0.05

A) Bar graph shows real time expression analysis of iNOS tran¬script in the ovarian tissues of rats of different experimental groups. B) Bar graph shows real time expression analysis of NQO1 transcript in the ovarian tissues of rats of different experimental groups. C) Bar graph is the representation of ovarian mitochondrial viability % in different experimental groups. D) Bar graph represents DNA fragmentation % in different experimental groups. Data of real-time PCR were used to calculate the relative expression of iNOS and NQO1 genes using the comparative threshold cycle method, and all values were normalized to the β-actin. The data represent means±SD (n=10/group). Cisplatin administration significantly increased ovarian iNOS (*P<*0.001), and DNA fragmentation % (*P<*0.001) and reduced NQO1 (*P<*0.001), and mitochondrial viability (*P<*0.001) compared to control group. RJ significantly decreased iNOS (*P<*0.001), and DNA fragmentation % (*P<*0.001) and increased NQO1 (*P<*0.001), and mitochondrial viability (*P<*0.001) compared to cisplatin group. a: indicates a significant difference when compared to the control group at *P<*0.05, b: indicates a significant difference when compared to cisplatin group at *P<*0.05. (RJ): Royal jelly, (iNOS): Inducible nitric oxide synthase, (NQO1): Quinone oxidoreductase 1

A) Mfn2 mRNA expression of different experimental groups. B) The most representative image of western blotting of ovarian Mfn2 protein level of different experimental groups. C) Mfn2 protein level in different experimental groups. D) The most representative image of western blotting of ovarian Bcl2 and Bax protein levels of different experimental groups. E) Bcl2 protein level in different experimental groups. F) Bax protein level in different experimental groups. G) Comparison of ovarian Mfn2, Bcl-2, and Bax protein levels in different experimental groups. The data represent means±SD (n=10/group). Our data showed that cisplatin significantly decreased Mfn2 (*P<*0.001), and Bcl2 mRNA expressions and protein concentrations (*P<*0.001) and significantly increased Bax mRNA expression and protein concentration (*P<*0.001) compared to control group. RJ restored Mfn2 mRNA expression and protein. Added to that, RJ re-established ovarian Bcl2/ Bax mRNA and proteins (*P<*0.001) compared to cisplatin group. a: indicates a significant difference when compared to the control group at P˂0.05, b: indicates a significant difference when compared to cisplatin group at *P<*0.05. (Mfn2): Mitofusin 2, (RJ): Royal jelly

## Conclusion

 Administration of RJ potently protects against mitochondrial-mediated ovarian damage by cisplatin via increasing the ovarian antioxidants, dropping pro-apoptotic protein Bax, stimulating anti-apoptotic Bcl2 and maintaining the integrity of mitochondrial membrane by activating Mfn2. Also, RJ is a potent reproductive stimulator by increasing the follicular count and ovarian activity. 
